# Where should Kirschner wires be placed when fixing patella fracture with modified tension-band wiring? A finite element analysis

**DOI:** 10.1186/s13018-019-1060-x

**Published:** 2019-01-11

**Authors:** Ming Ling, Shi Zhan, Dajun Jiang, Hai Hu, Changqing Zhang

**Affiliations:** Department of Orthopedic Surgery and Orthopedic Biomechanical Laboratory, Shanghai Jiao Tong University Affiliated Sixth People’s Hospital, No. 600 Yishan Road, Shanghai, 200233 People’s Republic of China

**Keywords:** Patella fracture, Modified tension-band wiring, Kirschner wire, Finite element analysis, Biomechanics

## Abstract

**Background:**

The position of Kirschner wires (K-wires) has an influence on the outcome of modified tension-band wiring (MTBW) in fixing patella fractures. However, the instruction for K-wires positioning is not clear enough. This study tried to clarify the effect of K-wires positioning and provide evidence for a more definite instruction.

**Methods:**

The sagittal position (SP) suitable for placing K-wires was evenly divided into SP 1–5 from anterior to posterior, and the finite element models of midpatella transverse fractures fixed by the figure-of-eight or figure-of-zero MTBW were built up at each SP. Separating displacement of the fracture, stress of the fracture, and stress of the internal fixations were measured at 45° knee flexion by using finite element analysis.

**Results:**

The separating displacement of the fracture was smaller at SP 3–5 (23% smaller than SP 1–2). From SP 1 to 5, the compression of the fracture surfaces increased (*R* = 0.99, *P* = 0.001); the improper stress area of the fracture surfaces decreased (*R* = − 0.96, *P* = 0.01), and so was the stress of K-wires (*R* = − 0.93, *P* = 0.02). However, the stress of stainless steel wires showed a stable trend.

**Conclusions:**

The SP of K-wires plays a role in the function of MTBW in the surgical management of transverse patella fracture. At 45° knee flexion, posteriorly placed (close to the articular surface) K-wires enable optimal stability and stress for the fracture, which provides basis for the positioning of K-wires in clinical practice.

## Background

The loosening of internal fixation is not rare among patients suffering from patella osteosynthesis. The interfragmental displacement was reported to be as high as 10–20% [1–3]. Furthermore, 2.4–12.5% of patients suffered from bone nonunion [4], and about 5% of patients underwent a second surgery [2]. As modified tension-band wiring (MTBW) is the recommended surgical technique for patella fractures [5, 6], especially the transverse type, the loosening related to it should be taken seriously.

MTBW is performed by drilling two Kirschner wires (K-wires) in a parallel fashion into the patella longitudinally and placing a stainless steel wire (SS-wire) anteriorly in the form of a figure-of-eight or figure-of-zero, which is a dynamic fixation system and supposed to convert the anterior tensile force into posterior compression force [5, 7]. The sagittal position (SP) of K-wires is one of the factors contributing to the complications. Hsu et al. [8] reported minor loosening of MTBW with anterior placement of K-wires in early postoperative stage, which might cause failure of fixation. While the SP of K-wires has not been intensively studied, it still needs to be proved in biomechanical studies and a bigger randomized controlled trial. Considering the clinical fact that various placements were made by different clinicians [8, 9], it is with great importance to guarantee that K-wires are placed at a proper SP.

This study compared the biomechanical characteristics of patellar MTBW under different sagittal positioning of K-wires by using finite element analysis and provided a basis for K-wires positioning.

## Methods

### Definition of the SP for K-wires

**Fig. 1:**
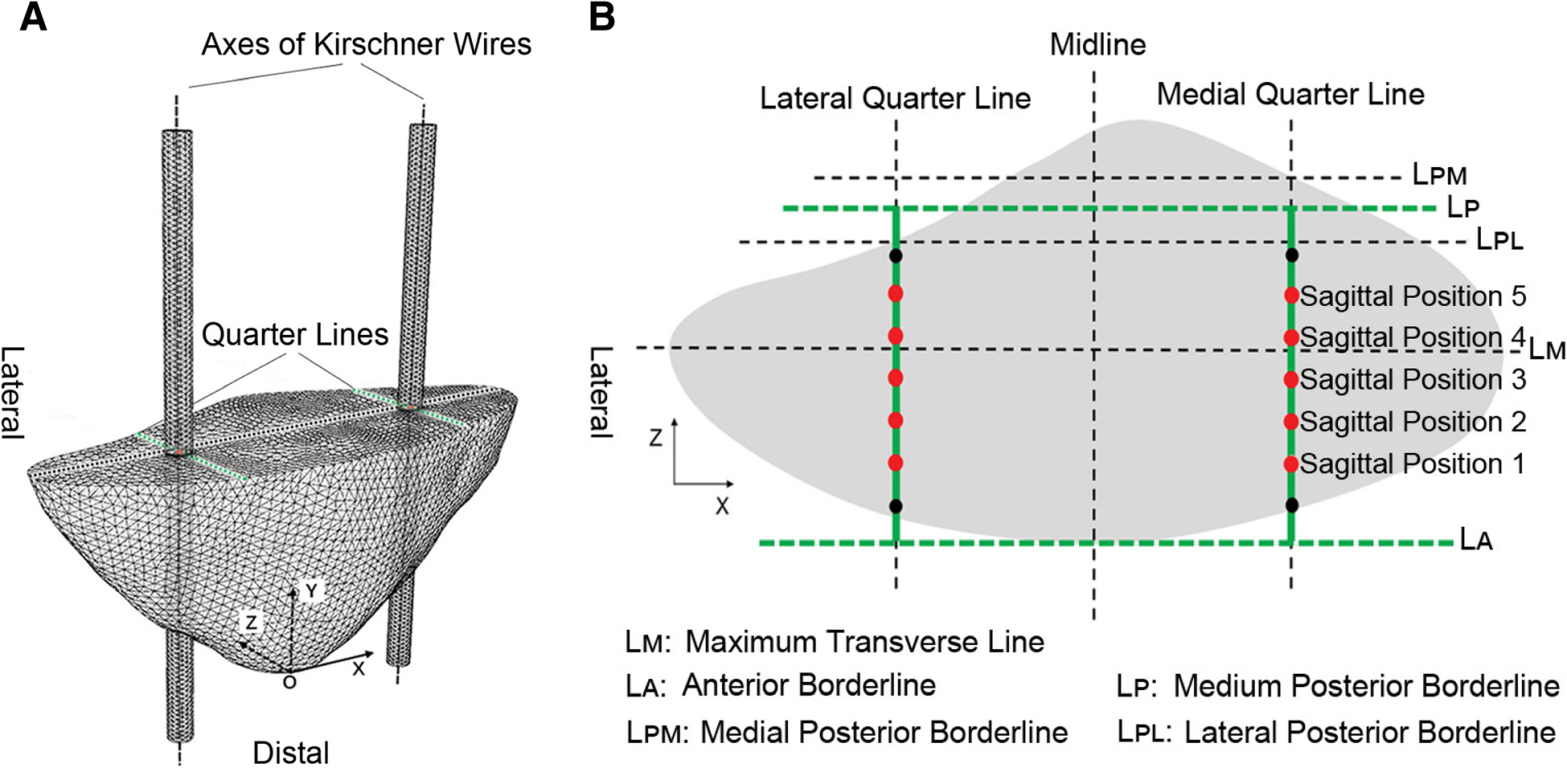
**a** The coordinate system of the patella. K-wires were placed in the direction of the *y*-axis (SP 3 for example). **b** Projection of all SPs on the median transverse section of the patella. SP 1–5 (red dots) were included in this study, and the most anterior and most posterior SPs (black dots) were excluded

The *x*-axis (from lateral to medial) was set parallel to the maximum transverse line connecting the medial and lateral edges on the median transverse section of the patella, and the *y*-axis (from distal to proximal) parallel to the line coinciding with the patellar crista. We defined the tip of the apex patella as the origin and established the coordinate system (Fig. 1a).

Two K-wires were placed in a parallel fashion along the direction of the *y*-axis and on the quarter lines of the median transverse section (Fig. 1a). Note that we focused only on the SP of K-wires (the direction of the *z*-axis) but not the coronal position (the direction of the *x*-axis). Thus, among all models, K-wires kept coinciding with the medial and lateral quarter lines, respectively on the AP view. SPs were determined by the following method. Three lines parallel to the *x*-axis were drawn on the median transverse section of the patella: the anterior borderline (along the anterior edge), medial posterior borderline (through the intersection of the medial quarter line and posterior edge), and lateral posterior borderline (through the intersection of the lateral quarter line and posterior edge). The medium posterior borderline was defined equidistant to the medial posterior and lateral posterior borderlines, and they were coplanar. The distance between the anterior borderline and medium posterior borderline was evenly divided by seven SPs. The most anterior and the most posterior SPs were excluded because they were too close to the margin, leaving five SPs named SP 1–5 for analysis (Fig. 1b).

### Finite element modeling

The imaging data was acquired from a healthy young male who had no history of knee joint pain or trauma. The slice thickness of CT scan (Siemens, Germany) was 0.6 mm, slice gap 0.6 mm, and resolution 512 px × 512 px.

**Fig. 2:**
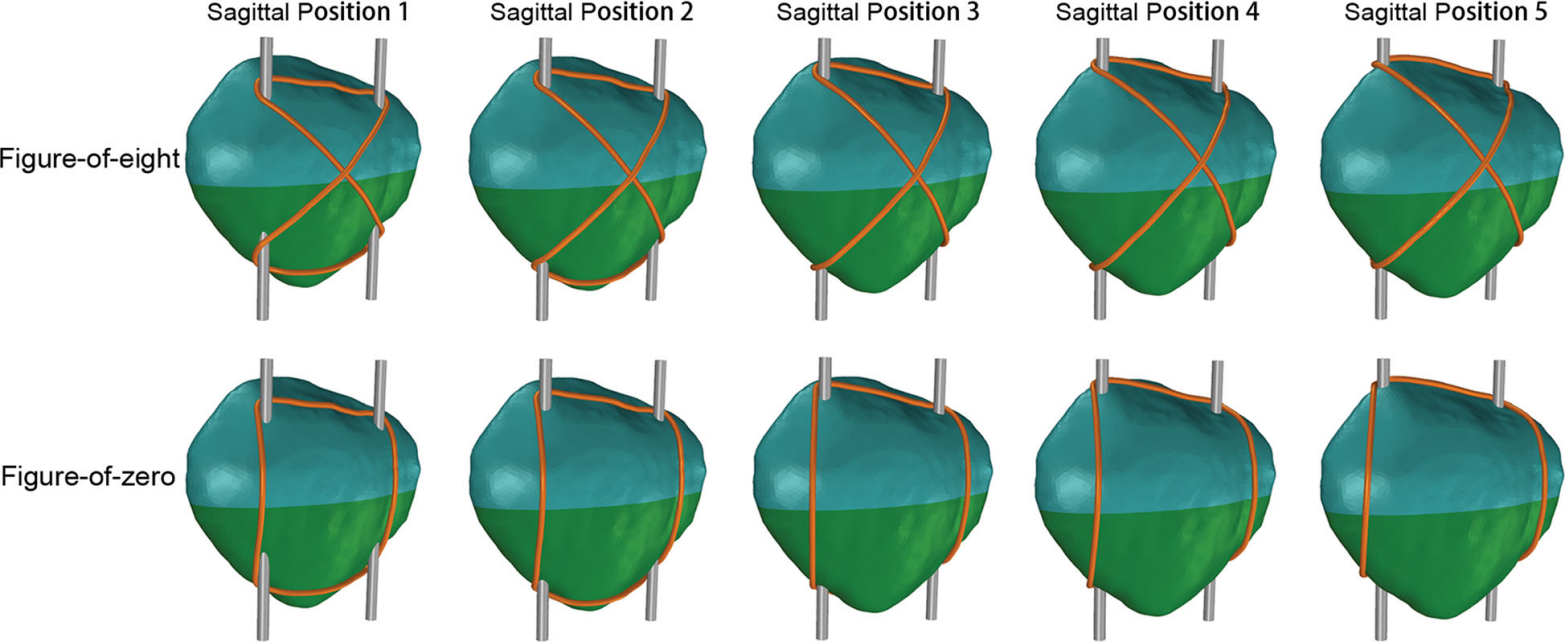
Patella fracture models with different SPs of K-wires and different fashions of wiring

Midpatella transverse fracture was created by intersecting the patella on the median transverse section. Two K-wires (2 mm in diameter) and a SS-steel wire (1 mm in diameter) were used to build up MTBW in the forms of a figure-of-eight and figure-of-zero respectively, and SS-steel wire was as close as possible to the bone [5, 7]. K-wires were at the same length in all models, and their exceeded parts would be removed in later stress processing. Considering that SS-wire would be firmer with a shorter total length, its path on the apex patellae was set as anterior path at SP 1–2 and posterior path at SP 3–5. We simplified the models without soft tissue and wire knots. All in all, there were 10 models (5 SPs × 2 fashions) for analysis (Fig. 2).

**Fig. 3:**
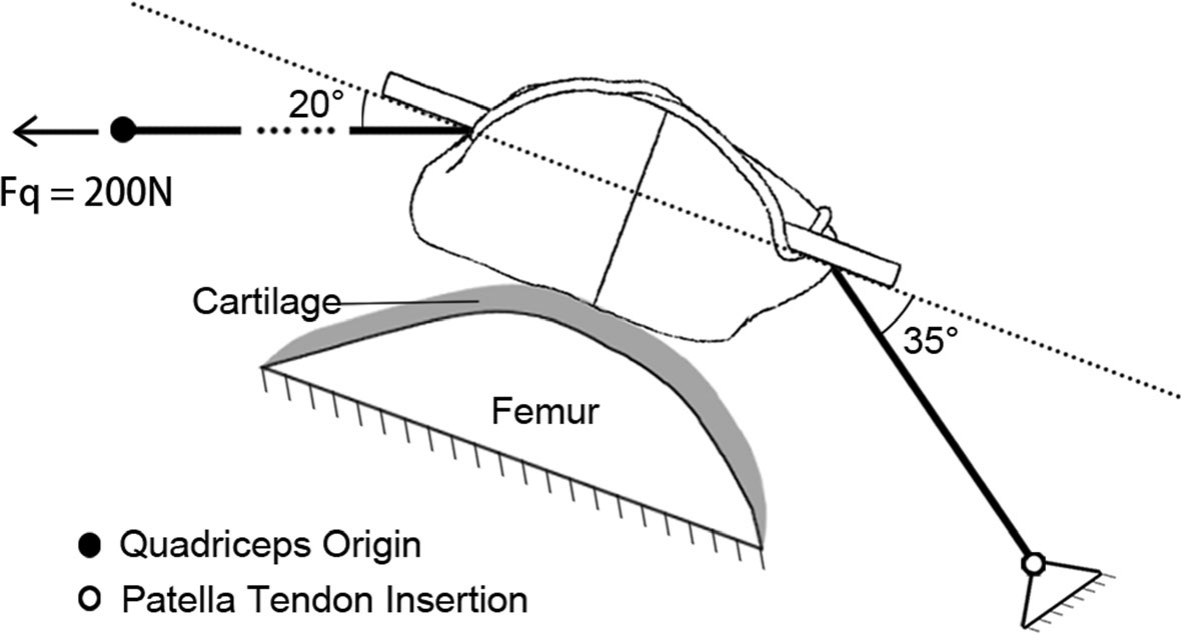
The model settings. The articular surface of the patella was in contact with the cartilage of the femur. The tendon attachments on the proximal and distal patella were coupled to the simulate origin of quadriceps (dot) and the insertion of patella tendon (circle) respectively. The origin of quadriceps was subjected to 200 N force along the direction of the muscle, and the movement of the femur was restricted, as well as the insertion of patella tendon. The model was open chained

The models were set in the condition of 45° knee flexion during non-weight-bearing extension with the middle part of the patellar articular surface contacting to a cartilage-covered distal femur. The attachment of quadriceps tendon and patella tendon on the patella were defined by CT and coupled to their simulate origin or insertion. The angle between the quadriceps tendon and patellar longitudinal axis was 20°, and that of patella tendon and patellar longitudinal axis was 35° based on previous biomechanical studies (Fig. 3) [10, 11]. In the simulation, the origin of quadriceps was coupled with a concentrated force of 200 N according to the published references [12] and coincided in the direction with the muscle. The femur and origin of the patella tendon were fixed. The material properties are shown in Table 1, among whom, patella was assigned according to the empirical expressions [13], the internal fixations [14, 15] and cartilage [16] were based on the published references, and the femur was treated as a rigid body to reduce the computational complexity. All materials were hypothesized to be isotropic and elastic without the consideration of plastic deformation. The Mimics 15.0 (Materialise, Belgium) was used for model construction, Rhinoceros 5.0 (Robert McNeel & Assoc., USA) for model design, and HyperMesh 14.0 (Altair, USA) for mesh optimization.

**Table 1:** The material properties

Parts	Specification	Element size	Material properties
Patella	40.1 mm in height, 44.5 mm in width, 19.1 mm in thickness, patellar ridge ratio 6:5	C3D4, 0.8 mm (0.4 mm in K-wires tunnels)	Based on the gray value distribution of distal femur*, elastic modulus 5–16.3 GPa, Poisson’s ratio 0.3
K-wire	2 mm in diameter	C3D4, 0.4 mm	Elastic modulus 200 GPa, Poisson’s ratio 0.3
SS-wire	1 mm in diameter	C3D4, 0.2 mm	
Femur	Larger than patellofemoral facet	C3D4, 2 mm	Rigid body
Cartilage	3 mm in thickness, cover femur side only	C3D4, 1 mm	Elastic modulus 7 MPa, Poisson’s ratio 0.47

*The empirical expressions: density = − 13.4 + 1017 × HU, elastic modulus = − 388.8 × 5925 × density. We used the expressions for femur because that for patella was not well validated

### Finite element analyses and statistics

The Abaqus 6.14 (Dassault Systèmes Simulia Corp., USA) was used for static simulation. In the simulation, the separating displacement and stress of the models were calculated. The total separation was defined as the difference between the average displacement of the elements on the proximal and distal fracture surfaces along the *y*-axis. The maximal and minimal separations were defined as the maximal and minimal separating displacement of the fracture surfaces along the *y*-axis. The average pressure of the fracture surfaces and the percentage of improper stress area (IPSA) on the fracture surfaces were calculated. In these IPSAs, the tensile stress is more than 0.15 MPa, which leads to fibrous tissue connection rather than osteogenesis [17, 18]. We used the layer of elements closest to the target objects to calculate the above parameters. In addition, the average stress of the internal fixations was calculated by averaging the von Mises stress of the elements of K-wires or SS-wires.

The values of all parameters were the means in all conditions, including the figure-of-eight and figure-of-zero fashions, except the comparison between them. The values of each fashion were calculated by averaging the parameters of all elements in condition of these fashions, respectively. For statistical analysis, we used the *t* test and Mann-Whitney *U* test for comparison, and linear regression for the linear correlation between the target parameters and SP. *P* values less than 0.05 were considered statistically significant.

## Results

**Fig. 4:**
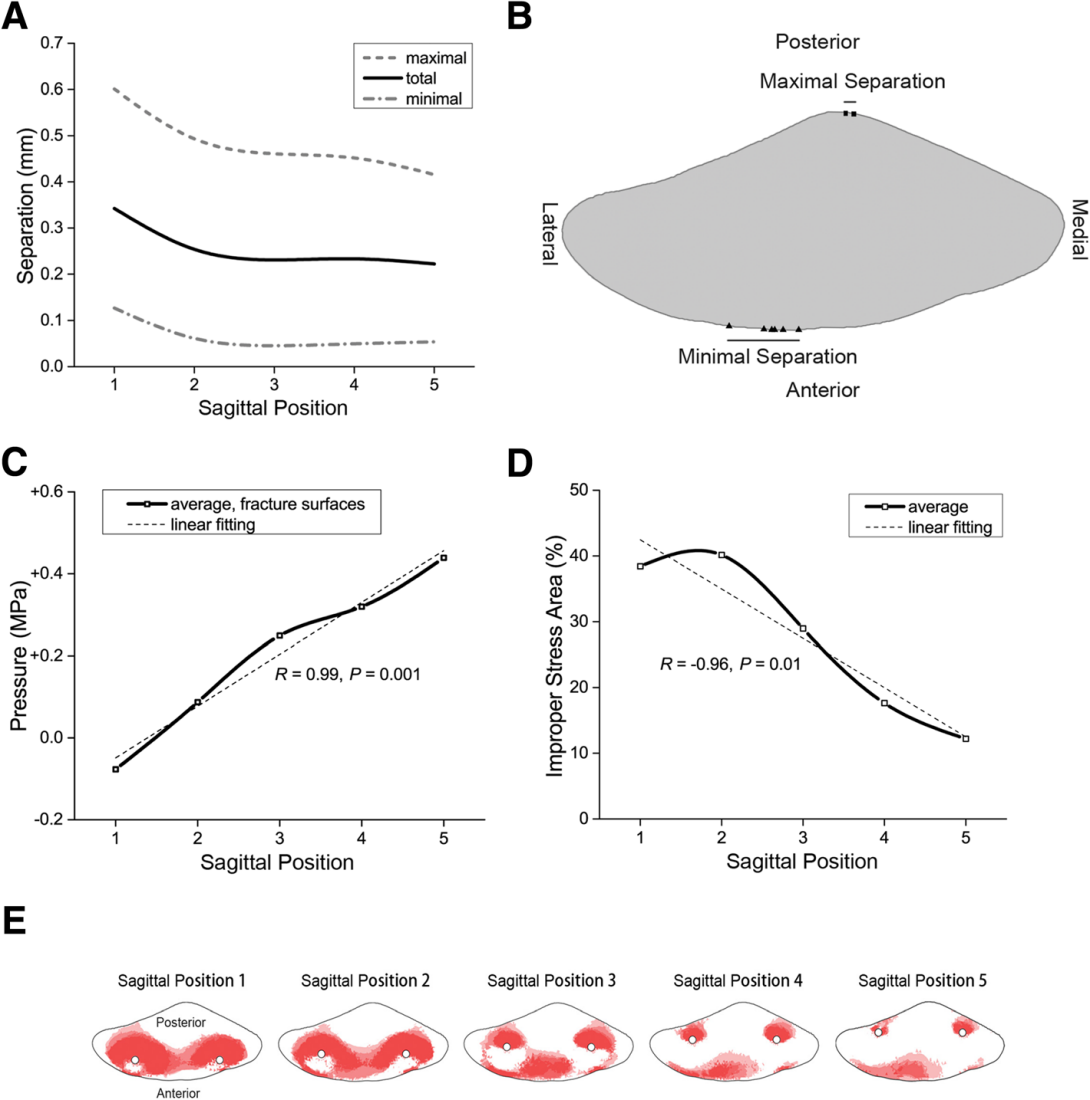
**a** The separation of the fractures at different SP. **b** The location of the maximal and minimal separation on the fracture surfaces. **c** The average pressure of the proximal and distal fracture surfaces. Linear with SP (*R* = 0.99, *P* = 0.001). **d** The IPSA% of the fracture surfaces. Linear with SP (*R* = − 0.96, *P* = 0.01). **e** The improper stress area of the fracture surfaces. Each figure was superimposed by four fracture surfaces (two fashions of wiring, two fracture surfaces of a model)

The average total separation was 0.26 ± 0.08 mm. It declined from 0.34 mm at SP 1 to 0.23 mm at SP 3 and maintained at about 0.23 mm at SP 3–5 (Fig. 4a). The average total separation at SP 3–5 was 23% smaller than SP 1–2. Note that smaller separation stands for more stability. The maximal separation ranged from 0.42 to 0.60 mm, the minimal separation ranged from 0.05 to 0.13 mm, and they occurred at the patellar crest and anterior lateral edge of the fracture, respectively (Fig. 4b).

The average pressure of the fracture surfaces was + 0.20 ± 0.20 MPa and increased linearly with the SP (*R* = 0.99, *P* = 0.001) (Fig. 4c), showing a compression effect of posterior positioning. Average IPSA% among all models was 27.47 ± 11.73%; it decreased from 40.15% at SP 1 to 12.22% at SP 5 and was linear with SP (*R* = − 0.96, *P* = 0.01) (Fig. 4d). The IPSAs of the fracture surfaces distributed mainly in the region posterior to K-wires and part of the anterior fracture surfaces, and posterior positioning of K-wires scattered the IPSAs (Fig. 4e).

**Fig. 5:**
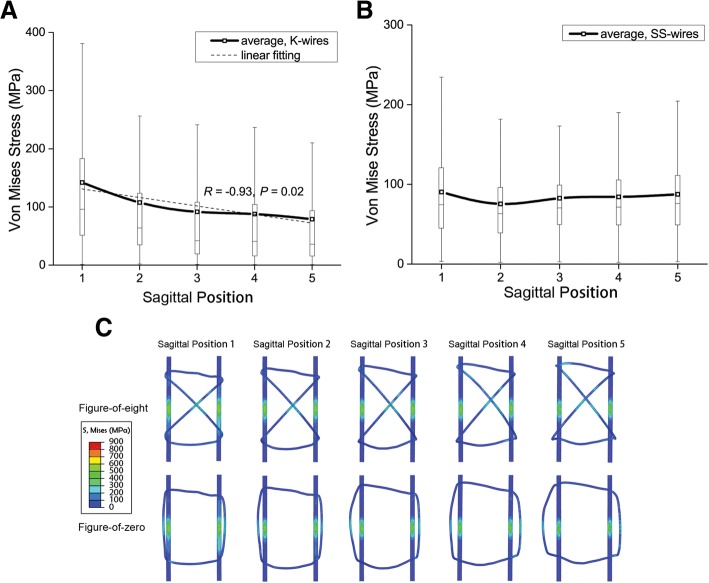
**a** The average von Mises stress of K-wires. Linear with SP (*R* = − 0.93, *P* = 0.02). The ends of the whiskers represent the boundaries of outliers, and it is the same in Fig. 5b. **b** The average von Mises stress of SS-wires. **c** The stress distribution on the internal fixations. The internal fixations were semi-transparent, including the stress on both anterior and posterior sides

The average von Mises stress of K-wires was 101.56 ± 22.73 MPa and linear with SP (*R* = − 0.93, *P* = 0.02); it decreased from 142.00 MPa at SP 1 to 78.83 MPa at SP 5 (Fig. 5a). The average von Mises stress of SS-wires was 84.06 ± 6.41 MPa and not in linear correlation with SP; it varied between 75.47 and 90.40 MPa with the minimum at SP 2 (Fig. 5b). The stress of K-wires was higher than that of SS-wires (*P* = 0.049) (Table 2). High stress appeared around the fracture surfaces and interaction regions of the internal fixations, and its impact area diminished gradually with SP (Fig. 5c).

**Table 2:** The comparison between the figure-of-eight and figure-of-zero wirings

	SP 1	SP 2	SP 3	SP 4	SP 5	Mean	Fashions comparison
Total separation (mm)							*P* = 0.008
Figure-of-eight	0.41	0.31	0.29	0.30	0.29	0.32	
Figure-of-zero	0.28	0.20	0.17	0.17	0.15	0.19	
Pressure (MPa)							*P* = 0.666
Figure-of-eight	− 0.10	+ 0.06	+ 0.23	+ 0.28	+ 0.40	+ 0.17	
Figure-of-zero	− 0.05	+ 0.12	+ 0.26	+ 0.36	+ 0.48	+ 0.23	
IPSA%							*P* = 0.617
Figure-of-eight	40.00	42.87	30.27	20.43	13.98	29.51	
Figure-of-zero	36.82	37.43	27.62	14.86	10.46	25.44	
Stress of K-wires (MPa)*							*P* = 0.625
Figure-of-eight	142.51	110.88	96.64	92.73	85.13	105.58	
Figure-of-zero	141.49	104.56	86.46	82.68	72.54	97.55	
Stress of SS-wires (MPa)*							*P* = 0.096
Figure-of-eight	95.01	76.62	84.58	89.46	92.40	87.61	
Figure-of-zero	85.80	74.33	80.58	79.08	82.78	80.51	

*SP* sagittal position, *IPSA* improper stress area

*The average von Mises stress of K-wires was significantly higher than that of SS-wires (*P* = 0.049)

The comparison of total separation between the figure-of-eight and figure-of-zero MTBWs revealed that the latter stabilize the fracture surfaces significantly (0.20 vs 0.32 mm, *P* = 0.008). However, there was no significant difference among the other parameters (Table 2).

## Discussion

MTBW for patella fracture, like a “hinge” at the tensile side, is able to neutralize distraction and tension forces and even converts them into compression when the knee joint flexes [5]. The quadriceps force and patellofemoral pressure are the main stresses in the knee joint motion. To counter them, MTBW must be tensile-resistant as well as bending-resistant [19]; SS-wires play the role of the former, and K-wires the latter. The ideal SP for K-wires recommended by the AO group lies in the center of the patella, approximately 5 mm below its anterior surface, and posterior placement is acceptable [5, 7]. However, this instruction depends largely on the surgical convenience and lacks biomechanical supports. As a result, different placements were made by clinicians due to the complexity of the fractures, as well as the ambiguity of the instruction of K-wires positioning. In this simulation study, we tried to clarify the effect of SP by taking biomechanics into account and provide evidence for a more definite instruction for K-wires positioning. As to the settings, we made some explanations as follows. We set the position at 45° knee flexion during non-weight-bearing extension. At this position, the contact area of the patella is roughly its middle articular surface [10], including the fracture line in this study; the patellofemoral pressure acts on the internal fixations directly, and the maximal separation of the fracture usually occurred [20]. Based on the knee joint kinematics, a 45° flexion angle is superior than the others for testing the validity of fixation in this study. At other flexion angles such as 0° or 90°, femur-tendon contact is the main contact form rather than femur-patella contact, thus the bending-resistance is not as critical as at moderate flexion. For this reason, the SP of K-wires might not be an important factor during slight and deep flexion.

The stability is of the main concern and presented by the displacement of the fracture surfaces. The separation in our study is in line with the study of Zderic et al. [12], in which the separation was 0.4 ± 0.3 mm. Also, our result consists with Hsu et al. [8]. They studied the SP of K-wires in 170 patients postoperatively by dividing the thickness of the patella into three equal segments on lateral X-ray films and found that the superficially placed K-wires contributed to minor loss of reduction, causing the failure of fixation probably. Thus, Hsu et al. suggested that K-wires should be placed in the middle third of the patella, whose counterpart in our study is approximately SP 3–5, but only the classification of SP was too simple. In our study, we made a detail classification for better biomechanical explanation. Claes et al. [17, 18] found that minor movement about 0.2 mm or more (no more than 1 mm) promotes healing. The separation of our study is consistent with the study of Claes et al., which indicated the efficiency of MTBW. What is noteworthy is that when factors such as the initial separation at the fracture surfaces, soft tissue inserting between SS-wires and the surface of patella, and slight looseness of the wire knots are taken into account, the actual separation could be larger. To ensure enough benefits for fracture healing, a smaller separation is better than a larger one. In our study, the separation of the fracture remained rather small and stable at SP 3–5; the IPSA% of the fracture surfaces and the biomechanics properties of K-wires improved linearly with SP. In our opinion, placing K-wires at a posterior SP enables enough stability for the fracture and provides more benefits in fracture healing.

Generally, posterior positioning of K-wires increases the exposure and difficulty of the surgery. Besides, it also increases the risk of soft tissue interposition, which would weaken the fixation of MTBW [21]. This may be the reason why the anterior placement of K-wires is not rare. To implement posterior positioning and get rid of complications, we suggest a clinician must first expose a posterior-enough positioning site without increasing invasion, second remove the barrier for placement, and third place K-wires as close to the articular surface as possible. It is notable that K-wires should not be placed in the anterior third of the patella.

MTBW takes effect by immobilizing the patella on the anterior surface, so the posterior separation was greater than the anterior one (Fig. 4b). With the K-wires being placed backwards, the stability of the posterior side was strengthened and smaller separation was demonstrated. The difference between the figure-of-zero and figure-of-eight MTBW is that the restriction of the former is on both sides of the anterior surface while that of the latter is in the middle. Thus, the figure-of-zero MTBW provided more stability for the fracture. Practically, when K-wires are placed at a posterior SP, SS-wire is close to the medial and lateral edge, and the risk of falling off is increased. Therefore, many clinicians prefer the figure-of-eight MTBW. Moreover, these fashions have different benefits for different fracture types, which could be validated in future studies.

There are some limitations. First, the models were simplified without soft tissue, wire knots, and the other tendons, and the plastic deformation of stainless steel was not considered. It is different from the practical application but would not influence the purpose to compare the SPs of K-wires. Second, we only chose the angle of 45° but no other angles for simulation. As we mentioned above, this angle is capable and superior for detecting the effect of SP of K-wires. In addition, too many results will be confusing and mislead the main purpose of this study. The other knee positions can be investigated as a supplementary in the future.

## Conclusions

The positioning of K-wires is dictated by many factors. This simulation study revealed that the SP of K-wires has an effect on the biomechanics of MTBW, and a posterior SP provides better stability and stress condition for the fracture at 45° knee flexion during non-weight-bearing extension. Posterior positioning (close to the articular surface) of K-wires should be made when possible. Clinical controlled trials and follow-ups are needed for validation.

## Abbreviations

IPSA: Improper stress area
K-wire: Kirschner wire
MTBW: Modified tension-band wiring
SP: Sagittal position
SS-wire: Stainless steel wire
